# Milk Sickness

**Published:** 1883-04

**Authors:** N. S. Townshend


### Art. XIV.—MILK-SICKNESS.

BY N. S. TOWNSHEND, M.D.

It is a common opinion that the affection known as Milk-sickness is caused by the use of milk or butter from cows that are the subjects of a disease called Trembles. This affection is fortunately so infrequent, and is restricted to such limited portions of the country that many regard the whole subject as mythical. Others who do not doubt that a serious and sometimes fatal malady, so designated, really exists, are still uncertain in regard to its nature, cause and appropriate treatment. On these points a few facts which came under the observation of the writer are here submitted:

*Symptoms of the Disease.*—A severe attack of Milk-sickness is characterized at the outset by persistent nausea and vomiting. A sense of chilliness is commonly experienced and great pain and tenderness are felt over the region of the stomach. After a time fever comes on; the tongue which was at first coated, and perhaps yellow, becomes dry and red, and sometimes cracks and bleeds. The vomiting continues, the matter ejected is at first bilious, but afterwards becomes dark like coffee-grounds, and there is obstinate constipation of the bowels.

*Nature of the Disease.*—The examination of cases where death occurred before relief could be obtaied, showed the disease to be an acute inflammation of the stomach, such as might have been produced by an irritant poison. The inner surface of the stomach was crimson red, with patches of darker color, or of a dull leaden hue. Evidences of inflammation, though in a less degree, were found in other portions of the intestinal canal.

*Cause of the Disease.*—That Milk-sickness, as it occurs in the human subject, is caused by using the milk from cows that are dieased or poisoned, or by eating the flesh of animals slaughtered while similarly affected, seems to be established by facts for which it would be difficult to find any other interpretation. Of a family of six persons, attended by the writer, five who had used the milk of the same cow, had Milk-sickness; the sixth person used neither milk nor butter and escaped entirely. At the time of the illness of this family a yoke of oxen belonging to them were sick with Trembles and both died. The cow which furnished the milk used by the family and which had pastured with the oxen was at the time severely sick, but finally recovered, perhaps because the poison was eliminated from her system with the lacteal secretion. Neither the family nor the cattle used other water than that taken from Lake

Erie. A thorough examination of the bodies of the oxen showed that both had died of inflammation of the stomach and bowels. This inflammation had been manifested during life by loss of appetite and especially by chills or rigors such as often precede inflammation. The cold or trembling stage in cattle is long continued, and constitutes the prominent feature of the affection, and hence the name *Trembles.* This case and others essentially similar leave little room to doubt that Milk-sickness is a direct consequence of using milk or other animal products that have in some way become poisonous. To the question, What produces such poisoning or disease of cattle, various answers have been given. Some have supposed the cattle to be affected with malarial fever, others have attributed the disease to bad water or poor pasture, and still others to some poisonous plant eaten by cattle with their food. The latter opinion, that Trembles is caused by a vegetable poison is generally accepted; but what is the plant which does the mischief? To this various answers have been made, one says it is *Rhus radicans* or Poison Ivy, another *Rhus toxicodendron*, or Poison Oak, etc. Probably the most satisfactory answer to the question is given in the Ohio Agricultural Report for 1858, in a communication from Mr. Vermilya, of Ruggles, Ashland Co., Ohio. In the same report it appears that Mr. John Rowe, of Fayette Co., had reached a similar conclusion many years previously. The results obtained by these gentlemen, and other observers to whom they refer, fix upon the *Eupatorium ageratoides* (sometimes called White Snake-root) as the cause of Trembles in cattle, and therefore indirectly of Milk-sickness in the human subject. In the report referred to a description and engraving of the plant are given. The *Eupatoriums* of which boneset or *Eupatorium perfoliatum* is a well-known example, are some of them very energetic in their action upon the animal economy, as any one who has taken boneset tea has doubtless experienced. The species known as *E. ageratoides* is among the most active of the family, and without doubt is sufficiently so to produce decidedly poisonous effects if taken in considerable quantity.

*Treatment of Milk-sickness.*—Regarding Milk-sickness, as simply an inflammation of the stomach set up by an irritant poison, the appropriate treatment is plain enough, but unfortunately not always successful, for in too many cases the poison has done its deadly work before the nature of the difficulty is understood or relief obtained. Usually the stomach has been relieved of its contents long before the arrival of the physician, whose endeavor then is to allay the nausea and vomiting, and arrest the inflammation. For such purpose, pounded ice should be given as long and as freely as it proves agreeable; a strong mustard poultice over the stomach will also be serviceable. As soon as the stomach will tolerate medicine, laxatives such as Seidlitz powders should be administered, and if the constipation is obstinate, injections containing a drop or two of Croton Oil may be required. Of the family already referred to, one was dead before medical aid could be obtained, a post mortem made plain the nature of the disease, and with the treatment above stated the other sick ones were promptly relieved.

*Conclusion.*—A more thorough knowledge of botany by farmers and veterinarians seems to be desirable. Structural and systematic botany are exceedingly interesting, and their study affords admirable mental discipling; but it is not sufficient to learn how plants grow, or how to name them correctly. The character of plants, their effect upon animal life, the uses to which they may be put, and the injuries they are capable of inflicting should be more thoroughly and generally studied. The *E. ageratoides* rarely grows in enclosed pastures, it is often found in woods and protected places, even there it is not abundant or widely distributed and cattle do not readily feed upon it, and are only induced to do so in seasons of drought when good herbage is deficient. Were the plant well known, a few hours would be sufficient to eradicate it entirely from any farm.

